# Influence of amorphous cellulose on mechanical, thermal, and hydrolytic degradation of poly(lactic acid) biocomposites

**DOI:** 10.1038/s41598-020-68274-x

**Published:** 2020-07-09

**Authors:** Wan Hafizi Wan Ishak, Noor Afizah Rosli, Ishak Ahmad

**Affiliations:** Department of Chemical Sciences, Faculty of Science and Technology, Universiti Kebangsaan Malaysia (UKM), 43600 Bangi, Selangor Malaysia

**Keywords:** Biopolymers, Polymer characterization

## Abstract

Eco-friendly materials such as poly(lactic acid) (PLA) and cellulose are gaining considerable interest as suitable substitutes for petroleum-based plastics. Therefore, amorphous cellulose (AC) was fabricated as a new reinforcing material for PLA biocomposites by modifying a microcrystalline cellulose (MCC) structure via milling. In this study, the mechanical properties, thermal properties, and degradability of PLA were analysed to compare the effects of both MCC and AC on PLA. The tensile and impact properties improved at an optimum value with AC at 8 wt% and 4 wt% fibre loading, respectively. Notably, a scanning electron micrograph analysis revealed improved AC fibre–matrix adhesion, compared with MCC fibre–matrix adhesion, as well as excellent interaction between AC and PLA. Both MCC and AC improved the hydrolytic degradation of PLA. Moreover, the biocomposites with AC exhibited superior degradation when the incorporation of AC improved the water absorption efficiency of PLA. These findings can expand AC applications and improve sustainability.

## Introduction

Pollution from slowly or non-degrading plastics has received increasing attention from developed countries, global organisations, and world leaders. In response, interest in the production of biopolymers as alternatives to petroleum-based plastics has been growing within the polymer community^1^. In recent years, the potential of biopolymers has been explored for the development of green composites, such as polybutylene-succinate, starch, and poly(lactic acid) (PLA)^2–4^.

Among these, PLA has been extensively studied owing to its sustainability, biocompatibility, and non-toxicity^5–7^. PLA is a thermoplastic polyester consisting of two different lactides (L&D) derived from the chiral nature of lactic acid. The polymerisation of the various lactides yields stereoisomers of poly(ʟ-lactic acid) (PLLA), poly(d-lactic acid) (PDLA), poly(d, L-lactic acid) (PDLLA), and poly(L-lactide-co-D, L-lactide) (PLDLLA)^8, 9^. PLA is obtained from the fermentation of simple sugars in agricultural waste to form lactic acid^10^. It has been utilised in diverse applications, including plastic packaging, automotive components, clothing, and medical equipment. However, its inadequate durability, thermal stability, degradation, and oxygen barrier properties have limited its use in specific applications, such as packaging^11,12^. Although PLA can be deemed a biodegradable polymer, it does not entirely fit in this category. In particular, recent findings have indicated that PLA has contributed to disposal problems^13–15^. This main drawback of PLA can be overcome by the introduction of natural reinforcing fibres, which will preserve the green properties of PLA.

Biopolymers reinforced with cellulose fibre have attracted significant interest owing to the excellent properties of cellulose, such as low density, affordability, excellent biodegradability, high specific strength, and promising sustainability^16,17^. Cellulose is the most abundant polymeric material and comprises both crystalline and amorphous regions^18^. The two regions can be extracted from semi-crystalline cellulose by different treatments. Acid hydrolysis can remove the amorphous region^19^, producing a highly crystalline material which is known as microcrystalline cellulose (MCC)^20,21^. Nevertheless, the different treatment variables and cellulose sources directly affect the degree of MCC crystallinity^22^. In previous studies, MCC was selected as a reinforcing material in the PLA matrix to preserve the PLA green properties as well as to enhance its mechanical, thermal, and biodegradability properties^20,23,24^. In those studies, MCC successfully improved the tensile modulus, thermal stability, and degradation properties. However, it also significantly reduced the tensile strength and elongation at break (EAB) of PLA. Therefore, attempts have been made to produce biodegradable PLA biocomposite without diminishing its green properties or its excellent mechanical properties.

To address these challenges, amorphous cellulose (AC) has been introduced as a reinforcing agent for PLA. AC can be produced via ball milling, hydrolysis of cellulose triacetate, and regeneration from cadmium ethylenediamine^18^. Among these methods, ball milling is a powerful and green method of inducing crystal structure modification of polymers. Unlike crystalline cellulose, AC is loosely arranged; thus, its mechanical properties and enzymatic hydrolysis rate, among other characteristics, differ from those of crystalline cellulose^18^. Compared with crystalline cellulose, AC is more flexible and more capable of interacting with the matrix owing to its loosely arranged structure. The use of AC alone as a reinforcement material to improve the toughness and degradability of PLA has not yet been exploited. The only study in which AC was used in biocomposite PLA was performed by Tsuboi et al.^25^ with the addition of three different PLA modifiers. Therefore, it is not comparable with the findings of our study. In addition, Cocca et al.^26^, who used AC as a reinforcing agent for poly(ɛ-caprolactone) (PCL), reported that PCL/AC biocomposites showed better mechanical properties than PCL/MCC biocomposites. Thus, attempts have been made to exploit the peculiar features of AC to study the interaction between AC and the PLA matrix.

The effect of the two different reinforcing materials, MCC and AC, on the properties of PLA biocomposites was the main objective of this study. The resulting biocomposites were evaluated via structural analysis, mechanical testing, thermogravimetric analysis (TGA), morphological analysis, water absorption testing, and hydrolytic degradation testing.

## Experimental

### Materials

PLA (Ingeo biopolymer grade 2003D; NatureWorks) had a molecular weight of approximately 120,000 g/mol. According to the manufacturer, it has a melt flow rate and density of 6 g/10 min (210 °C, 2.16 kg) and 1.24 g/cm^3^, respectively. The cellulose (Sigma-Aldrich) was in the form of microcrystalline powder with an average length and diameter of 90 µm and 51 µm, respectively.

### Preparation of AC

AC was prepared using a Fritsch Pulverisette 5 Planetary Mill. First, MCC was dried in a vacuum oven at 100 °C for 24 h. Then, the dried MCC was milled with 20 stainless steel balls with 10 mm diameters in 250 mL stainless-steel cups. The milling process was performed at a rotary speed of 450 rpm for 1 h.

### Preparation of PLA biocomposites

Before mixing, both AC fibres and PLA pellets were dried in an oven for 24 h at 100 and 40 °C, respectively. The mixing of PLA and AC was performed using a Haake Rheomix internal mixer (model R600). The desired amount of PLA pellets was placed in the mixer for 4 min with a rotor speed of 50 rpm and a temperature of 175 °C. Then, the AC was subsequently added with a variation from 0 to 10 wt%. For comparison, composites with the same MCC contents were prepared using the same procedures. After compounding, the biocomposite was pelletised using a crusher. Before moulding, the palletised samples were dried at 40 °C for 24 h. The palletised samples were compressed using a Labtech LP 50 hydraulic press at 3,447.38 kPa. First, the hot press was employed at 175 °C for 8 min, followed by the cold press for 15 min.

### Analyses and measurements

The structural properties of MCC and AC were characterised by X-ray diffraction (XRD) analysis. The investigation was conducted using a Bruker diffractometer (model AXS D8 Advance). The MCC and AC powders were subjected to a voltage and current of 40 kV and 40 mA, respectively. The analysis was performed under the radiation of Cu-Kα (*λ* = 0.1541 nm), and data were collected in 2*θ* between 10° to 50°.

Field-emission scanning electron microscopy (FESEM) was used to observe the differences in the surfaces, sizes, and shapes of MCC and AC. A Zeiss FESEM model Supra 55 VP was also used to study the impact-fractured surfaces of the prepared composites. The MCC, AC, and impact-fractured PLA biocomposites were coated with platinum and dried in an oven. The analysis was performed at a voltage of 3 kV. A PerkinElmer Fourier-transform infrared-attenuated total reflection (FTIR-ATR) spectrometer (model 2000) was used to record the spectra of the PLA biocomposites in the wave number range from 4,000 to 600 cm^−1^.

The mechanical properties of the PLA biocomposites were evaluated through tensile and Izod impact tests. These tests were conducted following ASTM D638 and ASTM D256, respectively. Seven samples from each biocomposite formulation were tested using an Instron 5,566 universal tensile machine at 16 °C. The tensile test was standardised using a 10 kN load cell, and the sample was stretched at 10 mm/min of cross-head speed. Meanwhile, the impact test was conducted using a Ray-Ran impact pendulum tester. The test was standardised using a 4.256 N hammer force at a velocity of 3.5 m/s.

The thermal stability and thermal history of the PLA and PLA biocomposites were investigated using a Mettler Toledo thermogravimetric analyser (models SDTA851e and DSC 882e, respectively). The samples were weighed, and thermogravimetric analysis (TGA) was performed from room temperature up to 600 °C under a nitrogen atmosphere. The heating rate of 10 °C/min and gas flow rate of 10 mL/min were selected for all the measurements. For differential scanning calorimetry (DSC) measurement, approximately 6–12 mg of PLA and PLA biocomposites were heated from room temperature to 200 °C. The analysis was performed at non-isothermal conditions at heating and cooling rates of 10 °C/min. The glass transition (T_g_), cold crystallisation (T_cc_), and melting (T_m_) temperatures were determined from first and second heating scans. The percent crystallinity (*X*_*c*_) was calculated as follows^27^:

1 $$X_{c} = \frac{{\Delta H_{m} - \Delta H_{cc} }}{{\Delta H_{m0} }} \times \frac{100}{w},$$

where ∆H_m,_ ∆H_cc_ and ∆H_m0_ = 93 J/g denote the enthalpy for melting, cold crystallisation, and melting for 100% crystalline PLA, respectively^28^. Here, *w* is the weight fraction of PLA in the sample.

The water absorption test was performed on the PLA biocomposite following ASTM D570. The sample was cut into a 20 × 20 mm dimension and dried in an oven at 100 °C for 24 h. The sample was weighed and then immersed in water. The next day, the sample was removed, dried, and weighed. The percentage of water absorption was calculated as follows^16^:

2 $$\% \;water\; absorption = ((M_{i} - M_{0} )/ M_{0} ) \times 100,$$

where *M*_i_ and *M*_0_ are the wet weight and initial dry weight of the sample, respectively.

In the hydrolytic degradation test, the sample was cut into the dimension of 30 × 15 × 0.3 mm. It was weighed and placed into capped flasks filled with 20 ml of distilled water at 37 °C for 15 days^29,30^. After 48 h, the specimen was removed and dried in an oven for 24 h at 100 °C and weighed. The weight loss was calculated as follows:

3 $$\% \;weight\; loss = ((W_{i} - W_{0} )/ W_{0} ) \times 100,$$

where *W*_i_ is its initial dry weight of the sample before the hydrolytic degradation test, and *W*_0_ is the weight of the sample after the hydrolytic degradation test.

## Results and discussion

**Figure 1:**
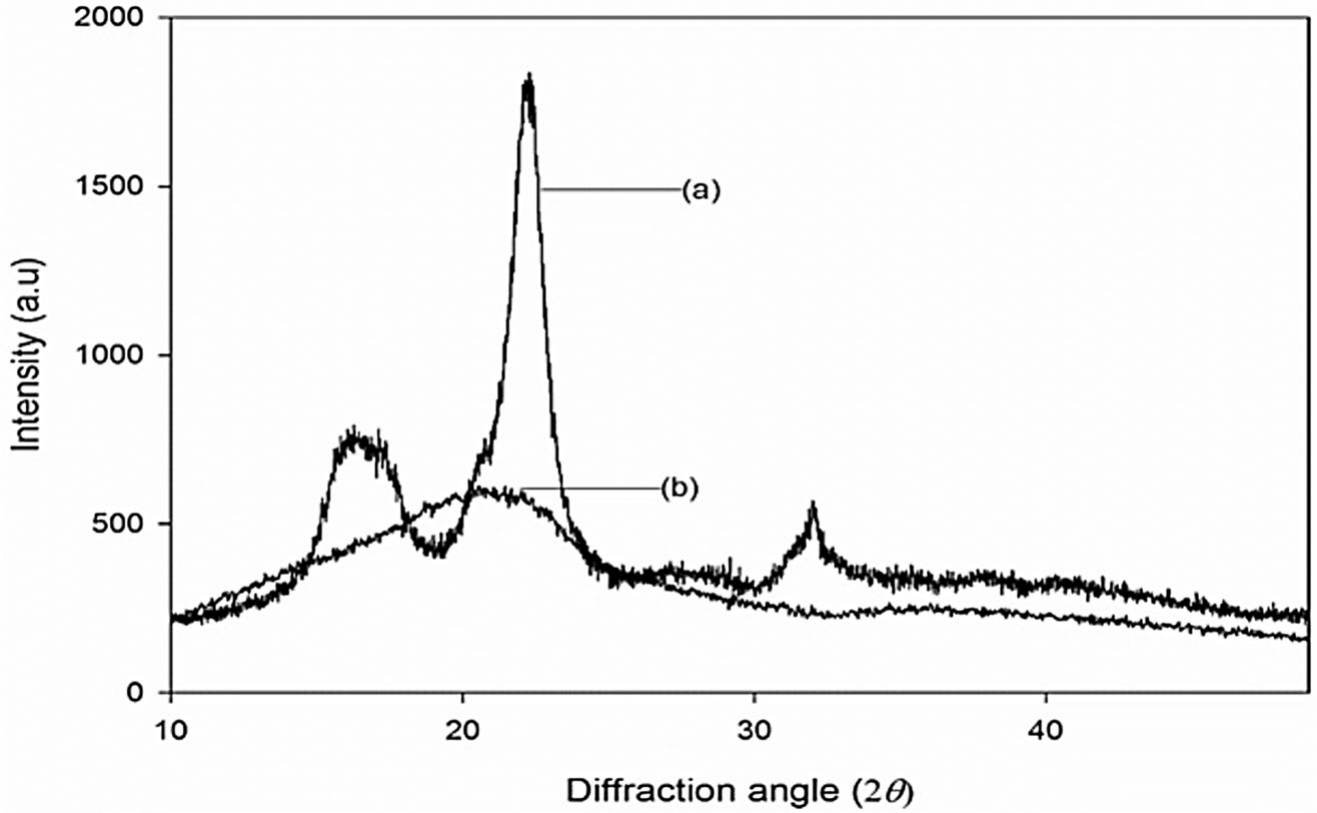
X-ray diffractograms of (**a**) MCC and (**b**) AC.

XRD diffractograms of the MCC and AC fibres are shown in Fig. 1. In Fig. 1a, typical diffractions due to cellulose I are observable at 2*θ* = 15°, 17°, 22°, and 32°, which correspond to (101), (10$\bar{1}$), (002), and (040), respectively^31^. After milling, these diffractions are no longer apparent. Alternatively, there is a broad peak at 2*θ* = 21° (Fig. 1b), which indicates that the cellulose I structure was transformed into an amorphous one. The significant XRD peak changes represent the formation of the amorphous structure after ball milling. The milling process weakened the hydrogen bonding in the crystalline part of the cellulose, substantially modifying it into an AC structure^32^. The same findings were reported by Zhang et al.^18^ and Avolio et al.^24^ Qualitatively, the changes in peak intensities and shapes, as well as the shifts, represent some changes of the crystalline structures in MCC. The incorporation of AC into the polymer matrix can result in the desired features of the biocomposite. In particular, the toughness of the composite may improve since the crystalline nature of cellulose is a lower priority in the newly fabricated material.

**Figure 2:**
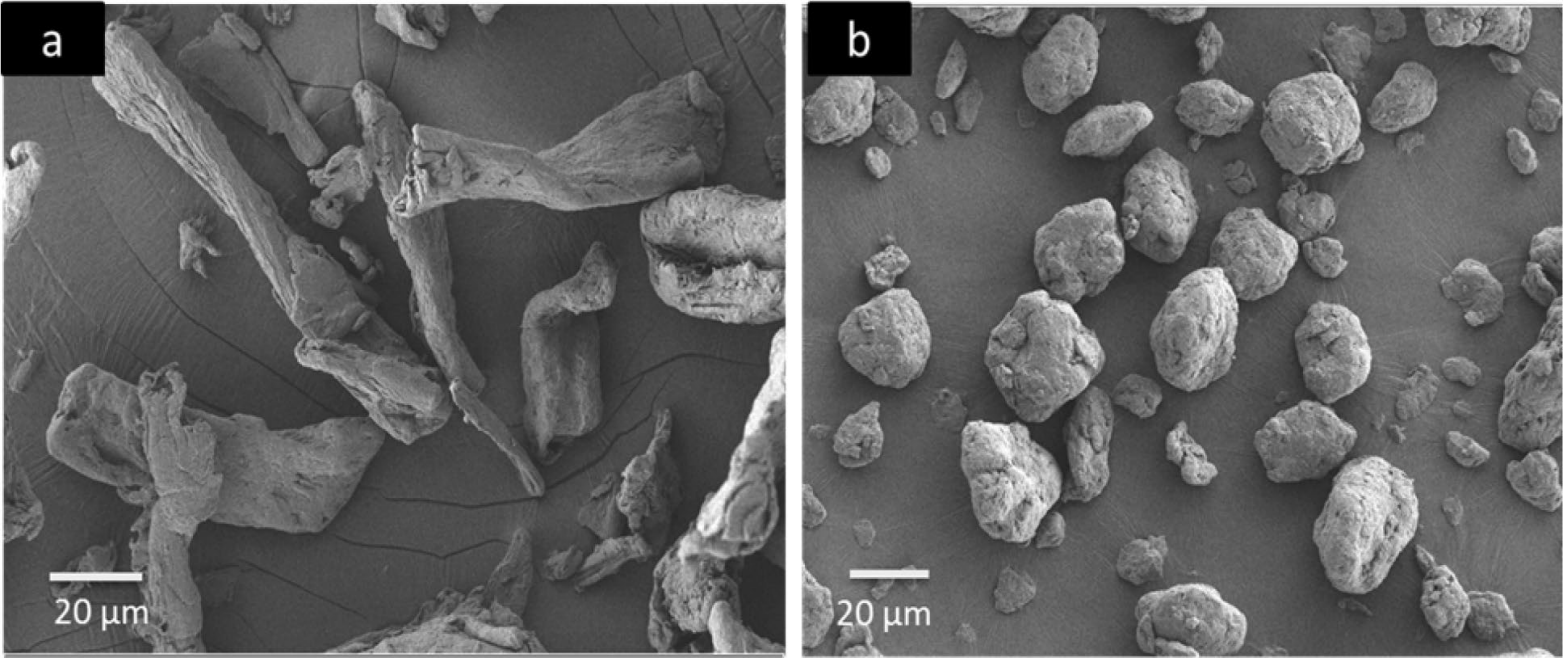
FESEM micrographs of (**a**) MCC and (**b**) AC.

The surface morphologies of MCC and AC are shown in Fig. 2. A morphological difference is evident in the long, fibrous shape of MCC compared to the circular or oval shape of AC. The circular or oval shape of the AC fibres indicates that the ball milling procedure effectively changes the MCC morphology. Moreover, MCC and AC exhibit different sizes: 93.30 ± 6.29 µm and 25.76 ± 1.32 µm, respectively. Thus, the ball milling procedure reduced the MCC fibre length and diameter, as reported previously^24^. The significant differences in the AC shape and diameter are expected to improve the dispersion of AC in the polymer matrix and consequently enhance the biocomposite performance.

**Figure 3:**
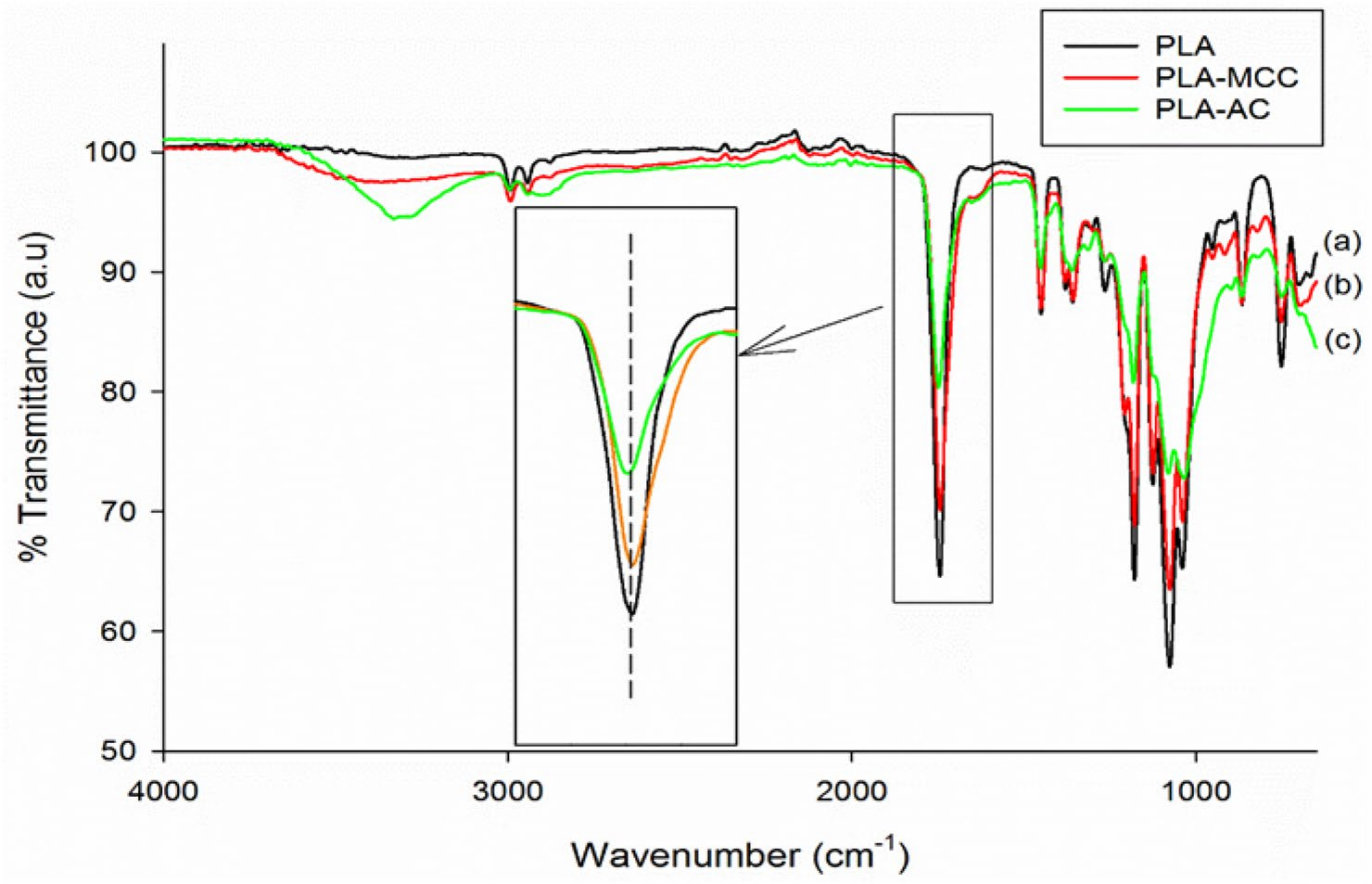
FTIR-ATR spectra of (**a**) PLA, (**b**) MCC biocomposite, and (**c**) AC biocomposite.

**Figure 4:**
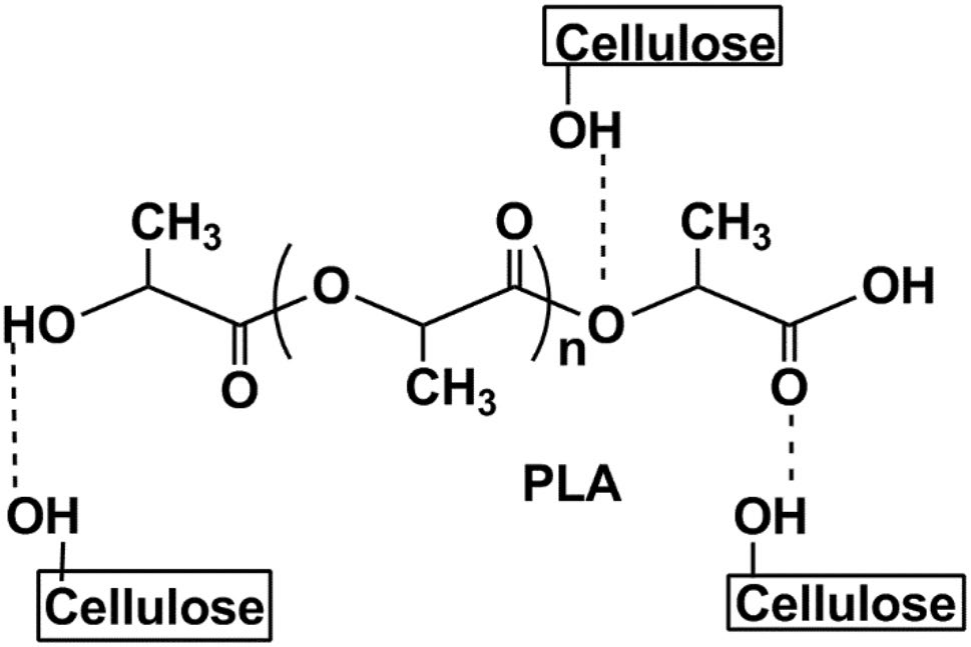
Probable interaction between PLA and cellulose.

FTIR-ATR analysis was performed to investigate the potential interaction between PLA with MCC, and PLA with AC. Figure 3 shows the intensity and peak shifting differences in the PLA-AC spectrum compared to the PLA-MCC spectrum. The most significant differences occur at 1744 cm^−1^ of the carbonyl (C=O) stretching vibration in PLA. It was previously reported that PLA can form hydrogen bonds with cellulose through its terminal hydroxyl and carboxyl groups^16^. The decrease in intensity of the C=O peak is more notable with the addition of AC than MCC. The difference in the intensity of the C=O peak suggests the chemical interaction that occurs between PLA and cellulose, as shown in Fig. 4.

**Figure 5:**
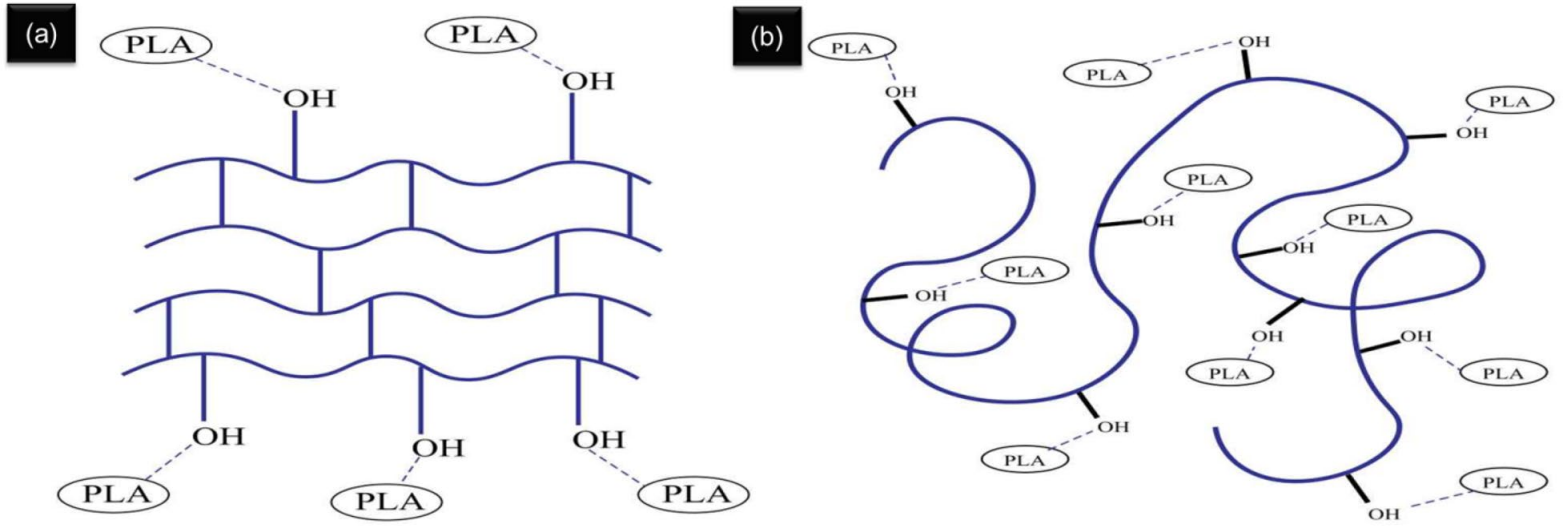
Schematic diagram of the interaction between (**a**) PLA with MCC and (**b**) PLA with AC.

Additionally, a peak shift is observable in the PLA-AC spectrum, whereas none is evident in the PLA-MCC spectrum. The shifting peak at 1744–1749 cm^−1^ in the PLA-AC spectrum suggests the occurrence of a chemical interaction. FTIR-ATR analysis shows that the chemical interaction between PLA and AC is more significant than that of PLA and MCC. The less-ordered structure of AC makes it highly capable of undergoing chemical interactions, which successively increases the development of hydrogen bonds with PLA. A schematic of the interaction between PLA with AC, and PLA with MCC, is illustrated in Fig. 5.

**Figure 6:**
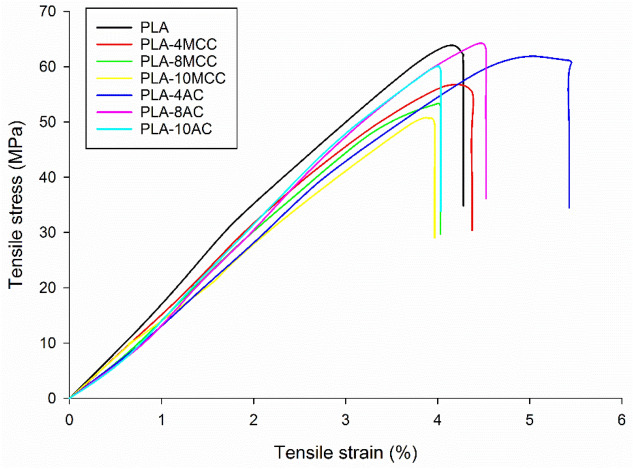
Tensile stress–strain curves of PLA, PLA-6MCC and PLA-6AC composites.

**Figure 7:**
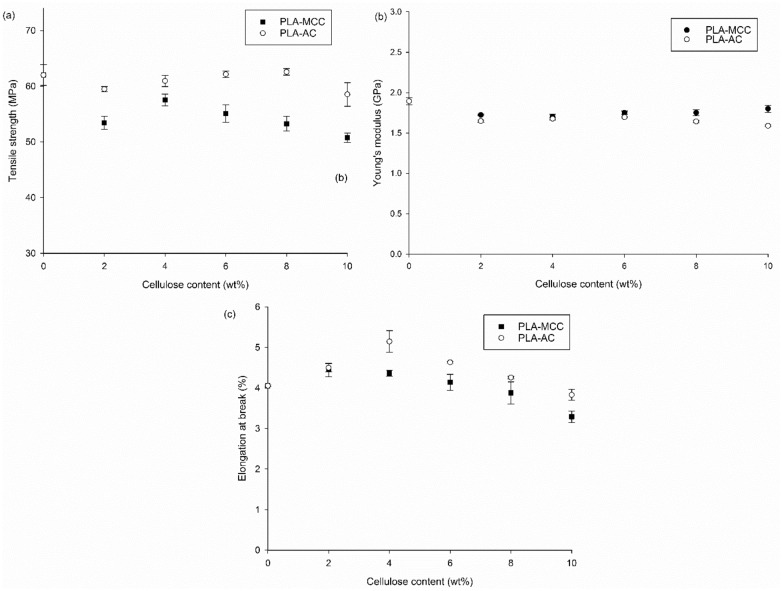
Tensile properties of MCC and AC biocomposites.

Figure 6 shows the typical stress–strain curves of the PLA and PLA biocomposites with different reinforcements. The stress–strain curve of PLA is slightly higher than those of the biocomposites. The PLA-AC stress–strain curve occurs between those of PLA and PLA-MCC. PLA displays a mean breaking strain of 4.0%, while PLA-4MCC and PLA-4AC show mean breaking strains of 4.3% and 5.1%, respectively. From the tensile stress–strain curve, various parameters, such as tensile strength (MPa), Young’s modulus (GPa), and elongation at break (%) of the composites were obtained and are plotted in Fig. 7.

The tensile strengths of the PLA biocomposites at different MCC and AC loadings are presented in Fig. 7a. The tensile strength of PLA shows no improvement with the incorporation of MCC. It is drastically reduced with the inclusion of 2 wt% MCC, then slightly improves to give the optimum value with the inclusion of 4 wt% MCC. At higher loadings of MCC fibres, the tensile strength further decreases. Specifically, the low tensile strengths of the composites may be attributable to the weak chemical interaction between MCC and PLA, as shown by the ATR spectra (Fig. 3). Moreover, the stress transfer from PLA to MCC also suffers owing to this weak interaction and adhesion. Accordingly, more applied load is carried by MCC, thereby reducing the MCC inefficiency for load bearing and leading to the low biocomposite strength. The same observation was reported by Haafiz et al.^20^ In their study, the introduction of MCC decreased the tensile strength of PLA by up to 30% with the addition of 5 wt% MCC.

In addition, the further decrement in tensile strength beyond the optimum MCC loading (4 wt%) is caused by the inhomogeneous distribution of fibre, which subsequently promotes agglomeration. Furthermore, high MCC loading causes the presence of high fibre ends, which promote the debonding between the matrix and fibre on account of the high-stress concentration^16,33^. In contrast, the tensile strength shows an insignificant change with the inclusion of 2 wt% AC, followed by an increment to the optimum value at 8 wt% AC, and a decline with the further addition of AC. While the difference in tensile strength of PLA-AC biocomposites to PLA is insignificant, it is noteworthy to compare with the PLA-MCC biocomposites. The improvement of the tensile strength through the addition of AC fibres may be attributed to the chemical interaction between AC and PLA through the formation of hydrogen bonds between cellulose and PLA^34–36^. The variations of the interactions between AC and PLA and between MCC and PLA are visible in the ATR spectra (Fig. 3).

The stress transfer from PLA to AC is also improved owing to the formation of hydrogen bonds and good adhesion between PLA and AC. Furthermore, the improvement of the tensile strength of the PLA-AC biocomposites could be attributed to the shape and sizes of AC. The almost circular or oval shape of AC improves the fibre dispersion. Moreover, the total surface area increases with the decreasing fibre sizes, thereby increasing the tensile strength of the biocomposites^37^.

The incorporation of both MCC and AC decreases the stiffness of the PLA materials, as observed from the slight decrement of Young’s modulus values for PLA in Fig. 7b. This suggests that the AC and MCC particles can move freely within the PLA chain. The PLA chain segments also can move freely when provided with adequate space to move, thereby reducing Young’s modulus. However, Young’s modulus of the PLA-MCC composite increases with the fibre loading. Meanwhile, Young’s modulus of PLA-AC decreases with the increasing fibre loading. The low Young’s modulus of the PLA-AC biocomposite compare to the PLA-MCC biocomposite could be due to the high occurrence of the chemical interaction between PLA and AC. As the chemical interaction between PLA and AC increases, the coalescence among the C = O group of PLA weakens and consequently reduces the stiffness^38,39^.

Figure 7c shows the EAB of the MCC and AC biocomposites with different fibre contents. The EAB improves slightly with the inclusion of 2–4 wt% MCC and is reduced with the further addition of MCC fibres. Previously, the reduced EAB with the incorporation of MCC to PLA was also reported^20,23,40^. From those studies, it was revealed that the incorporation of MCC significantly reduced the EAB of PLA^20,23,40^. Notably, the incorporation of AC successfully increased the EAB of PLA; the EAB values for the AC biocomposites at all fibre loadings were higher than that of the matrix alone. This improvement could be explained by the diminished rigidity of the amorphous cellulose structure compared with that of the crystalline cellulose structure. The EAB increase could also be described by the formation of hydrogen bonds between PLA and AC.

**Figure 8:**
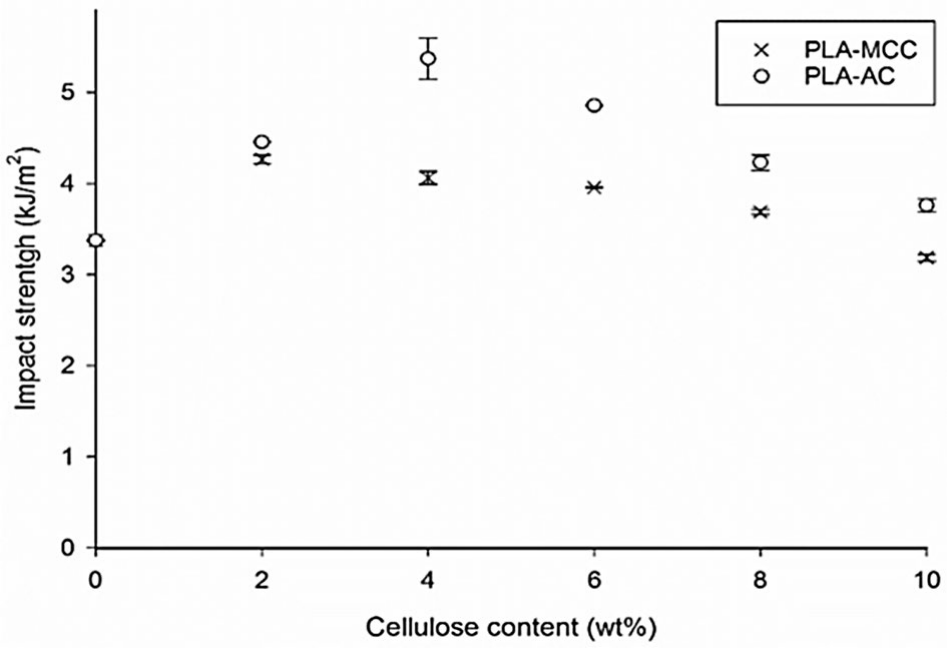
Impact strength of MCC and AC biocomposites.

The impact strengths of the PLA-MCC and PLA-AC biocomposites are presented in Fig. 8. Although the impact strengths of both the MCC and AC biocomposites increase upon the addition of fibres, they exhibit different trends. Notably, the MCC biocomposites exhibit the optimum impact strength at 2 wt% MCC. The increase at this loading could be attributed to the poor adhesion between MCC and PLA. The poor adhesion could promote fibre pull-out during the impact test and consequently increase the impact strength^41^. Further addition of MCC fibres caused the impact strength to decrease, although it remained higher than that of the matrix alone.

In contrast, the AC biocomposites show increasing impact strengths with optimum impact strengths at 4 wt% AC. These characteristics can be clarified by the increase in the chemical interaction between PLA and AC, leading to the improved adhesion between the AC fibres and PLA (Fig. 3). However, further addition of AC fibres caused the impact strength to decrease, although it remained higher than that of the matrix alone. The agglomeration of AC within the PLA matrix could explain this behaviour. The high accessibility of amorphous material for the chemical interaction not only resulted in fibre–matrix bonding but also caused high fibre–fibre bonding. This bonding caused the fibres to agglomerate and be dispersed non-uniformly, decreasing the impact strength.

**Figure 9:**
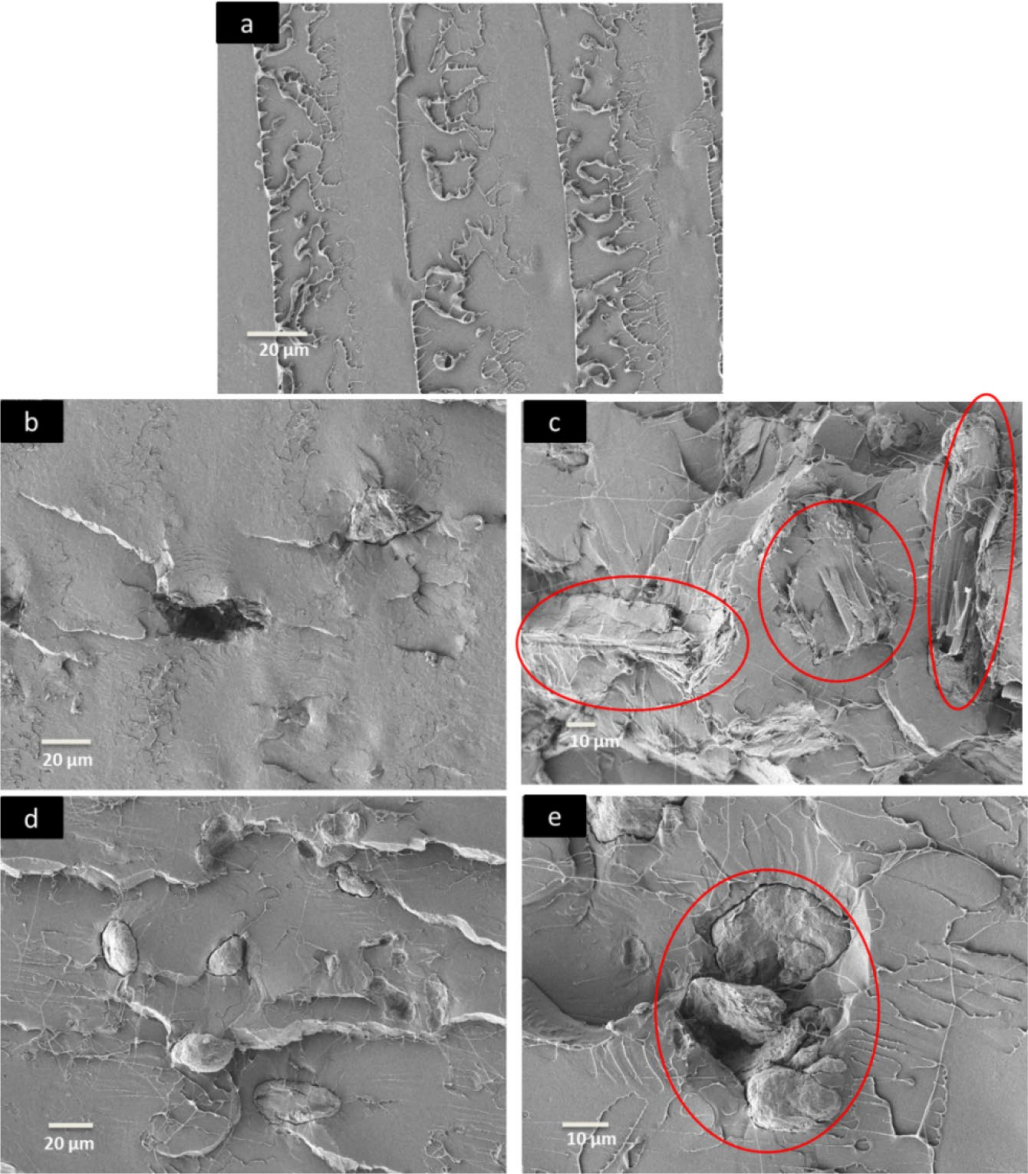
SEM micrographs of the impact fracture surfaces of the (**a**) PLA, (**b**) PLA-2MCC biocomposite, (**c**) PLA-10MCC biocomposite, (**d**) PLA-2AC biocomposite, and (**e**) PLA-10AC biocomposite.

The impact-fractures surfaces of the PLA, PLA-MCC biocomposite, and PLA-AC biocomposite are shown in Fig. 9. The micrographs of PLA and its biocomposites show morphological differences: The PLA fracture surface is smooth, whereas the biocomposites with added MCC and AC fibres exhibit increased roughness. Figure 9b shows a micrograph of a 2 wt% MCC biocomposite fracture surface, which exhibits a pulled-out fibre with sufficient fibre dispersion. This characteristic suggests weak interaction between the MCC and PLA. Increasing the numbers of fibre pull-outs and the formation of numerous holes within the matrix provide evidence of the improved impact strength of the PLA-2MCC biocomposite. These two phenomena could increase the impact strength because the pull-out mechanism requires high energy, and the voids can help the dissipation of energy during impact propagation^41^. However, the factors that increase the impact strength, such as the fibre pull-outs, also contribute to the decrease in tensile strength. Figure 9c shows that the fibre breakage dominates the fibre pull-out on the rough surface of the PLA-10MCC biocomposite. Consequently, the fibre breakage mechanism dominates the energy dissipation during the impact and thus decreases the impact strength.

The 2 wt% AC biocomposite shows a fracture surface rougher than those of the 2 wt% MCC biocomposites (Fig. 9d). Following the impact, the AC fibres remain intact within the PLA matrix, suggesting excellent adhesion. The improved of AC dispersion and fewer pulled-out fibres at low loadings further illustrate the tensile strength improvement of the PLA-AC biocomposites. From the same micrograph, some debonding between the fibre and matrix is observable. The debonding of PLA and AC could be contributed to the improvement in impact strength as it lowers the sensitivity of the biocomposite toward cracks and notches^42^. The rough fracture surface of PLA-10AC also reveals the agglomeration of AC, as shown in the micrograph. The addition of an excessive amount of AC beyond the optimum fibre loading leads to the formation of fibre–fibre bonding, resulting in the agglomeration of fibres.

**Figure 10:**
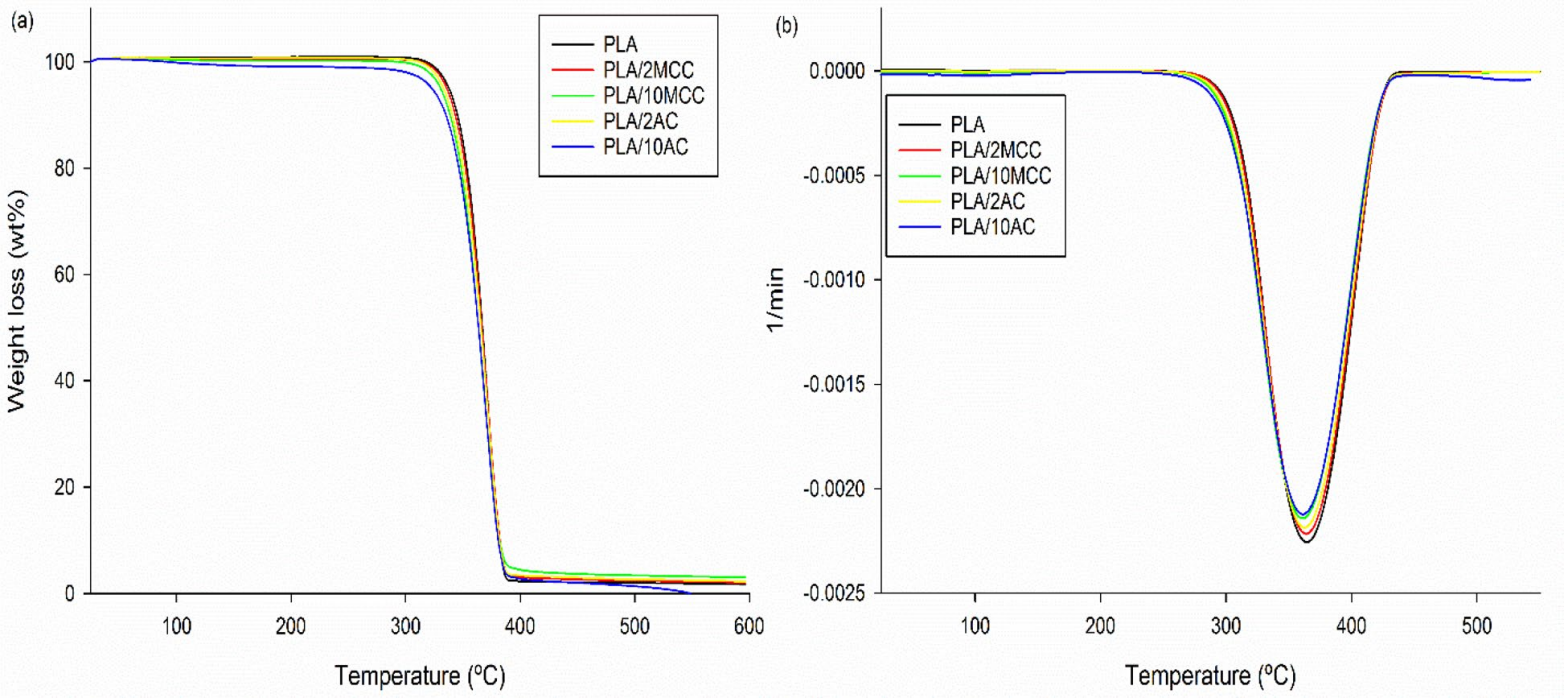
(**a**) TGA, and (**b**) derivative thermogravimetric (DTG) thermograms of PLA and PLA biocomposites.

The thermal stability of PLA, the PLA-MCC biocomposite, and the PLA-AC biocomposite are shown in Fig. 10. A slight weight loss (< 2 wt%) at ~ 100 °C is attributed to the evaporation of moisture. The thermal stability of PLA insignificantly decreases with the addition of both MCC and AC. The maximum temperature decreases by approximately 2 and 3 °C with the incorporation of 10 wt% MCC and AC, respectively, confirming that AC and MCC insignificantly affect the PLA thermal degradation.

**Figure 11:**
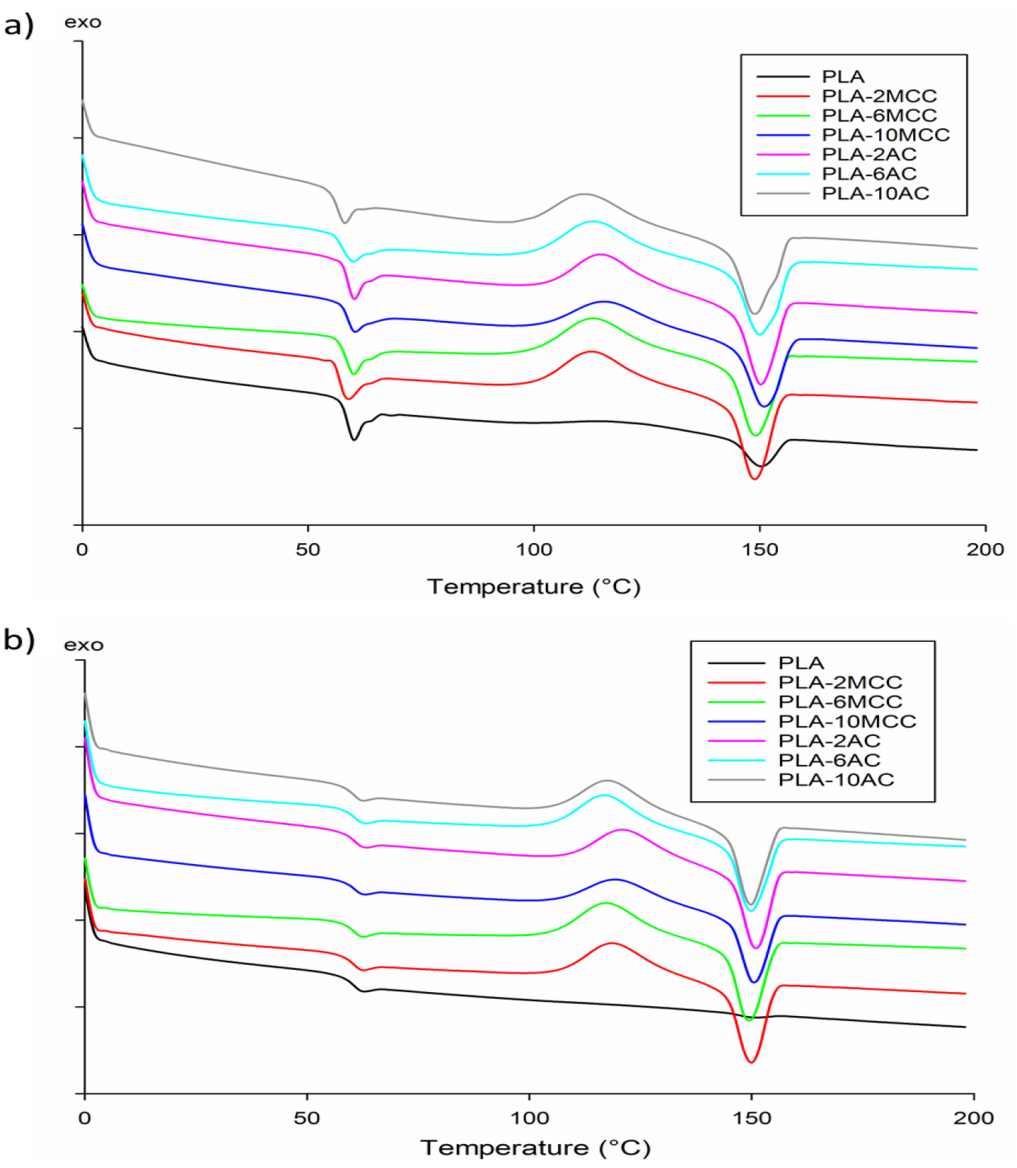
DSC (**a**) first heating and (**b**) second heating scan thermograms of PLA and PLA biocomposites.

DSC analysis was conducted to obtain information on the phenomena of T_g_, T_cc_, and T_m_, as well as the degree of crystallinity of PLA and its biocomposites. The obtained thermograms during the first and second heating scan are summarised in Fig. 11a, b, respectively. Meanwhile, the detailed of thermograms are recorded in Table 1. From the DSC curves, PLA exhibits T_g_ of 60 °C and T_m_ of 150 °C without the appearance of a T_cc_ peak. The addition of MCC does not significantly affect T_g_ and T_m_ of PLA; they remain the same during the first and second heating scan. Likewise, T_g_ and T_m_ of PLA are unaffected with the addition of AC. However, during the first heating scan, T_g_ of PLA decreases slightly up to 2.4 °C with the addition of 10 wt% AC. The T_g_ values are supported by the Young’s modulus value shown in Fig. 7b, where the incorporation of MCC and AC does not significantly affect the properties. Meanwhile, the slight reduction of T_g_ with the incorporation of 10 wt% AC is evident by the lowest value recorded for Young’s modulus. The difference in T_g_ values during the first and second heating is expected as it directly depends on the thermal history^39^. Glassy material is formed as a result of rapid cooling, which increases the values of T_g_ during the second heating^43^.

**Table 1:** Thermal characteristics and the degree of crystallinity of PLA and its biocomposites.

Samples	First heating scan			Second heating scan			*X*_c_ (%)
	T_g_ (°C)	T_cc_ (°C)	T_m_ (°C)	T_g_ (°C)	T_cc_ (°C)	T_m_ (°C)	
PLA	60.2	–	150	62.2	–	150.3	7.8
PLA-2MCC	59	112.5	148.6	62.2	118.5	149.4	10.1
PLA-6MCC	60	112.6	148.8	62.2	116.6	149	12.2
PLA-10MCC	60	115.2	150.6	62.2	119.4	150.1	13.1
PLA-2AC	59.8	115	149.9	62.2	125	150.6	13.0
PLA-6AC	59.6	112.9	149.6	62.2	116.4	149.4	11.4
PLA-10AC	57.8	111	143.6	62.2	117.2	149.4	10.5

In terms of the T_cc_ phenomenon, no appearance of T_cc_ in the PLA DSC thermogram is due to the low crystallisation ability of PLA grade 2003D compared to the other PLA grade^44^. The presence of both MCC and AC promotes PLA crystallisation, which indicates that both MCC and AC can act as a nucleating agent for PLA. T_cc_ slightly increases owing to the increased MCC content. Meanwhile, the rising of the AC content decreases T_cc_ up to 8 °C. The crystallisation occurs rapidly at low T_cc_ and results in less perfect crystal, while the high value of T_cc_ suggests that formation of a perfect crystal is owing to the PLA ability to crystallise^39,45^. With that ability, the degree of crystallinity also increases with the addition of MCC and AC. The degree of crystallinity of PLA (7.8%) increases up to 13% with the incorporation of 10 wt% MCC and 2 wt% AC. The value of percent crystallinity is supported by the T_cc_ value, where the high value of T_cc_ forms a perfect crystal, thereby, increasing the degree of crystallinity.

**Figure 12:**
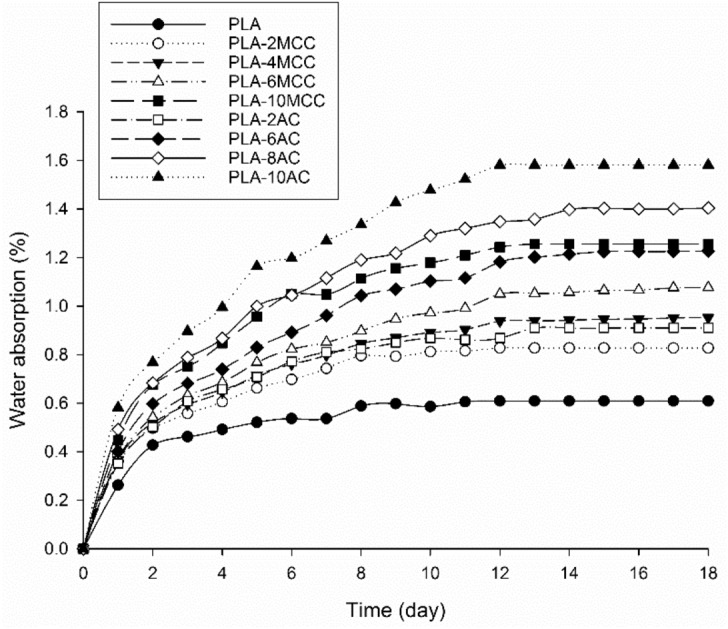
Percentages of water absorption for the PLA and PLA biocomposites.

One of the most fundamental properties of PLA biocomposites is water uptake, especially when the biodegradability is a critical feature in the application. Water absorption indirectly determines the hydrolytic degradation of PLA^13^. Figure 12 shows the percentage of water uptake by PLA, PLA-MCC, and the PLA-AC biocomposites as a function of time. The same observation patterns are observed in all samples. Additionally, the water absorption percentage of PLA increases with the MCC and AC contents, with the PLA-AC biocomposite possessing the maximum percentage. The high water absorption could be due to the hydrophilic nature of cellulose. The high hydrophilic nature of cellulose enables it to interact easily with water molecules. Cellulose will lose hydrogen bonds on the interfaces between themselves and compensate by forming new hydrogen bonds with water molecules^46^. Thus, the high cellulose contents increase the formation of hydrogen bonds and consequently cause the percentage of water absorption to increase. The increased water absorption of the AC biocomposites can be explained by the randomly arranged structure of AC, which facilitates water penetration. In contrast, the uniformly arranged structure and high crystallinity of MCC limits water penetration, resulting in low water absorption.

**Figure 13:**
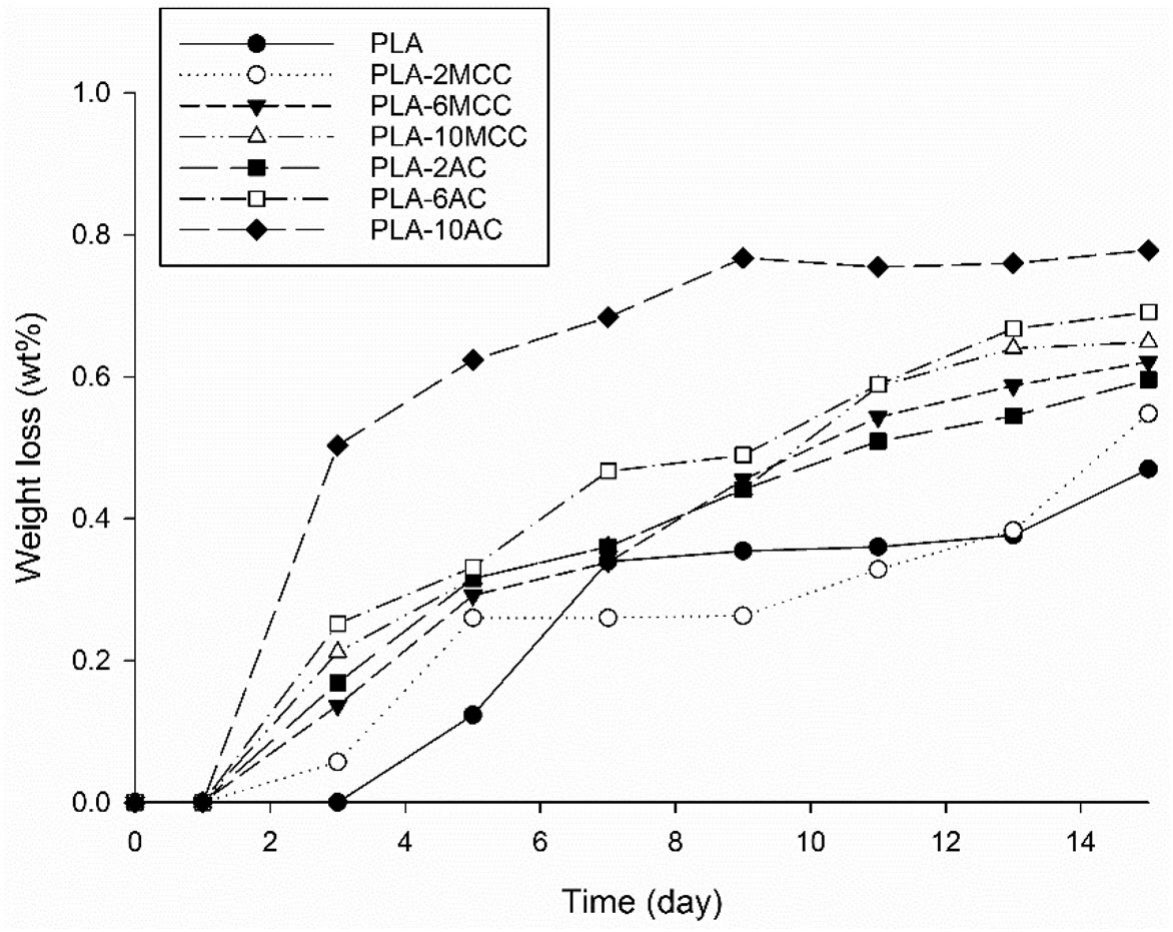
Weight loss of PLA and PLA biocomposites after hydrolytic degradation.

Figure 13 shows the effect of MCC and AC on the hydrolytic degradation of PLA as a function of time. All the samples begin degrading at day 3 except PLA. Notably, the hydrolytic degradation rates of PLA increase with the incorporation of MCC and AC. It is well known that PLA degrades by simple hydrolysis, which depends on the moisture content, molecular weight, crystallinity, molar mass, temperature, and water absorption^47,48^. The degradation of PLA was found to occur at a temperature above its glass transition temperature (T_g_ ~ 50–60 °C)^11^. In this study, the test was standardised at 37 °C to provide a more realistic environment. Furthermore, this study focused on the relation between water absorption and the degradation of PLA, as water is necessary for hydrolytic degradation. Previously, it was revealed that the biodegradability of PLA and its biocomposites mainly depends on the percentage of water absorption^16,49^. That finding explains the low hydrolytic degradation of PLA compared to its biocomposites in the present study.

The biodegradability of PLA improved with the incorporation of MCC and AC, where the PLA-AC biocomposites lost more mass than the PLA-MCC biocomposites. The high mass losses of the AC biocomposites can be explained by the randomly arranged structure of AC, which facilitated the penetration of water for hydrolysis. The mass loss increased as the content of AC and MCC increased. The improvement could have been due to the high water absorption from the high hydrophilicity of cellulose. Therefore, the hydrolytic degradation exhibited similar trends to that of the water absorption trends shown in Fig. 12. Another possible explanation is the adhesion between the cellulose and the PLA matrix: the poor adhesion may have served as the primary path for water entering the matrix^49,50^. In the present study, the fibre–matrix interface was not sufficient for high cellulose loading and subsequently increased the total interface surface. Consequently, the degradability of the composites increased.

## Conclusion

A new green composite of PLA-AC with various compositions of reinforcing AC was successfully fabricated in this study. Notably, incorporating AC within PLA yielded extraordinary performance in terms of the studied mechanical properties: It does not only improve the toughness but also increases the impact strength of the PLA biocomposites. Additionally, the hydrolytic degradation of the PLA-AC biocomposites showed definite improvement as the AC composition increased. Confirming this finding, the water absorption test demonstrated a more significant increase in the hydrolytic degradation of PLA-AC biocomposites. The improved properties of PLA-AC biocomposites are attributable to the unique properties of AC, particularly its loosely ordered arrangement and circular shape, as indicated in the XRD and morphology discussions, respectively. In conclusion, the enhancement of PLA properties through the incorporation of AC could further promote the use of AC in other industrial applications.
